# Case Report: Antiviral treatment after pterygium surgery based on corneal confocal microscopy

**DOI:** 10.3389/fmed.2026.1817461

**Published:** 2026-04-28

**Authors:** Fangfang Jin, Wenwei Li, Ting Wu, Bin Wang

**Affiliations:** 1 Tongde Hospital of Zhejiang Province Affiliated to Zhejiang Chinese Medical University (College of Integrated Traditional Chinese and Western Medicine Clinical Medicine), Hangzhou, Zhejiang, China; 2 Department of Ophthalmology, Tongde Hospital of Zhejiang Province, Hangzhou, Zhejiang, China

**Keywords:** corneal confocal microscopy, corneal diseases, herpes simplex keratitis, herpes simplex virus, pterygium

## Abstract

**Introduction:**

Pterygium is a chronic inflammatory condition that commonly occurs on the bulbar conjunctiva of the nasal palpebral fissure. Its etiology is multifactorial, and surgical excision remains the primary treatment method. However, recurrence and other complications may affect treatment outcomes. Currently, the diagnosis and treatment of secondary corneal diseases following pterygium excision surgery vary. Therefore, *in vivo* confocal microscopy of the cornea holds significant clinical importance in clarifying the etiology of the disease.

**Case presentation:**

An elderly diabetic patient presented with redness and swelling at the medial canthus of the eye and was diagnosed with recurrent pterygium. Following pterygium excision, follow-up examinations at 3, 10, and 20 days post-operation revealed a favorable prognosis. Approximately 1 month post-surgery, an anterior segment photograph of the eye showed an annular defect near the lateral limbus, which was considered to be “marginal keratitis”. Treatment with immunosuppressive eye drops was initiated. After 3 days of medication proved ineffective, a follow-up consultation was sought. An anterior segment photograph with staining revealed a geographic superficial ulcer on the temporal side of the cornea, with no significant stromal infiltration. Through corneal confocal microscopy, a partial defect in the left eye’s corneal epithelium was detected in the lesion zone. This was characterized by irregular, speckled, highly reflective spots and the presence of inflammatory cells. Additionally, there was a noticeable absence of subepithelial nerve fibers, along with activated Langerhans cells and stromal cells that were hyperactive in the periphery, exhibiting patchy and uneven deposits. Fortunately, the peripheral area displayed a sufficient number of endothelial cells. Following antiviral treatment, the patient’s ocular condition improved.

**Conclusion:**

Herpes simplex keratitis (HSK) is a rare but serious complication following pterygium surgery. This study confirms the evidence of secondary viral infection after pterygium surgery. In suspected cases, in addition to routine examination, conducting aetiological investigation is essential for early diagnosis and guiding treatment, thereby achieving a good visual prognosis.

## Introduction

Pterygium is a chronic inflammatory lesion, named for its similarity to the wing of an insect, often occurring on the bulbar conjunctiva in the nasal canthal region of the palpebral fissure (1, 2), accompanied by neovascularisation. The etiology of pterygium is caused by multiple factors, among which increased ultraviolet radiation exposure has a significant correlation with the development of the disease (3). When a pterygium grows and invades the cornea, it can cause corneal astigmatism, leading to visual impairment, which may affect one or both eyes (4). Surgical resection remains the primary method for treating pterygium (5). Common surgical methods include simple pterygium excision, pterygium excision combined with limbal autologous stem cell transplantation, and amniotic membrane transplantation (6). Although generally effective, post-operative complications can significantly affect prognosis and treatment success rates. Postoperative common complications include recurrence, symblepharon, corneal ulcer, scleromalacia, fibroma, and corneal opacity. This study uses corneal confocal microscopy to ascertain the etiology of corneal complications secondary to pterygium excision, thereby demonstrating its significant clinical importance.

## Case description

**Figure 1:**
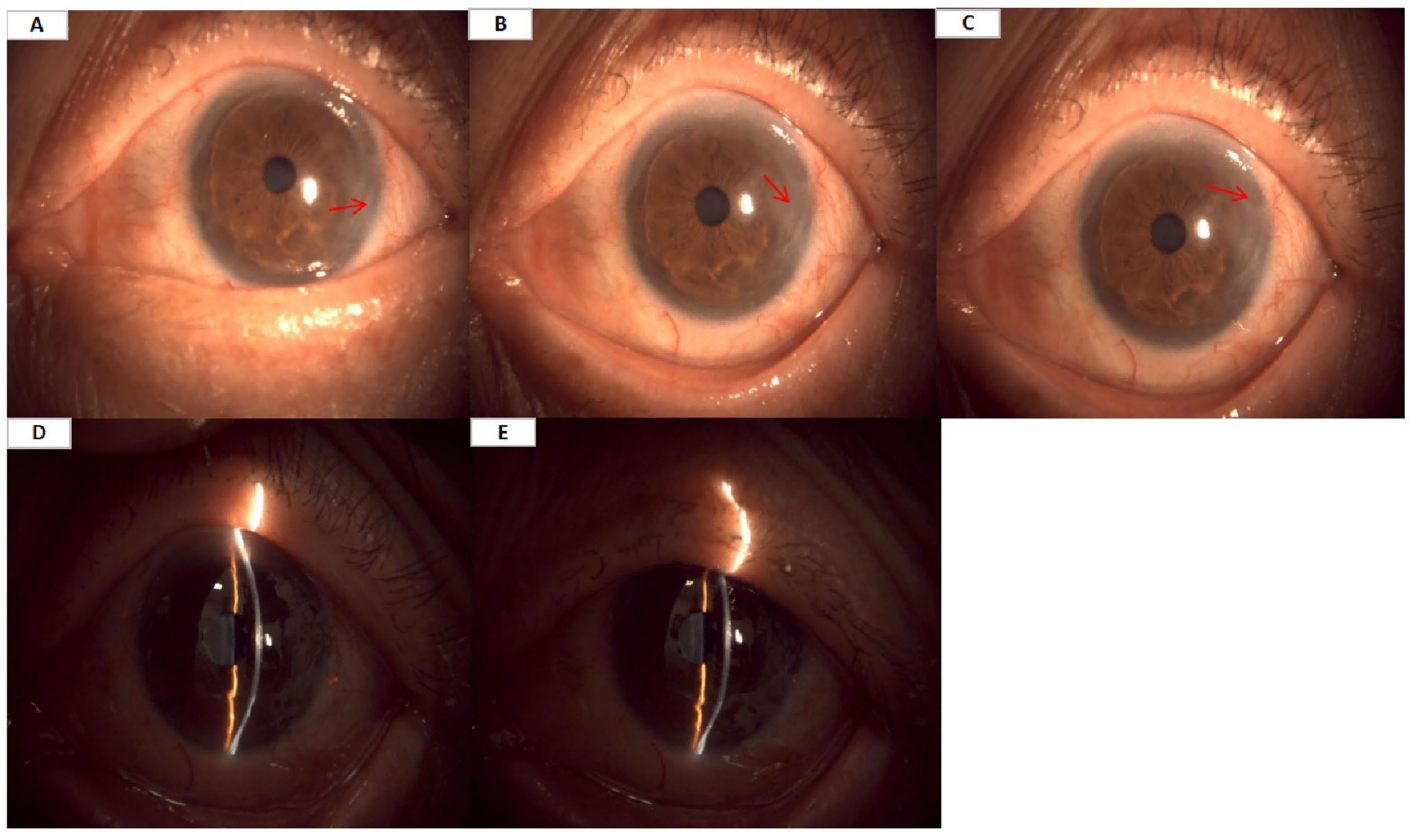
**(A–C)** At one-month follow-up after left eye pterygium excision, anterior segment photography of the left eye showed a ring-shaped defect near the lateral canthal limbus of the cornea. **(D,E)** Central cornea being transparent.

A 71-year-old Chinese male patient presented to our hospital’s ophthalmology department due to redness and swelling in the medial canthus of the left eye for more than 3 years. This patient has a history of over 10 years of type 2 diabetes mellitus (T2DM) with poor glycemic control, but currently shows no signs of diabetic complications. Three years ago, he underwent a bilateral pterygium excision. Over the past 3 years, the patient had experienced subjective redness and swelling at the lateral canthus of the left eye, with a progressive enlarging trend. The patient attended our hospital for a follow-up visit after left eye pterygium surgery, Physical examination showed right eye vision of 0.5, left eye vision of 0.4, intraocular pressure of 10.5 mmHg in the right eye and 11.0 mmHg in the left eye. Slit-lamp examination revealed mild conjunctival congestion in both eyes, pterygium proliferation in the temporal corneal area of the left eye, slight punctate staining of both corneas, and lens opacity. The preliminary diagnosis was “left eye recurrent pterygium,” with no other significant abnormalities were found in the examination, and surgical treatment was recommended. Consequently, under local anesthesia on August 28, 2025, a “left eye pterygium excision with autologous conjunctival transplantation” was performed. The patient was subsequently treated with a compound preparation of tobramycin and dexamethasone eye drops (4 times daily for 3 days), 0.1% pranoprofen eye drops (4 times daily for 3 days), and a compound preparation of tobramycin and dexamethasone eye ointment (1 time at night for 3 days). On the 3rd postoperative day, slit-lamp examination of the left eye revealed eyelid edema, conjunctival congestion, intact sutures, corneal macula, and limbal edema, the remaining condition was as preoperative, and tobramycin dexamethasone eye drops (4 times daily for 8 days), 40,000 IU recombinant human epidermal growth factor eye drops (4 times daily for 8 days), and a compound preparation of tobramycin and dexamethasone eye ointment (3 times daily for 3 days) were administered. Subsequently, a follow-up examination was conducted 10 days postoperatively, and clinical signs showed mild eyelid edema and conjunctival congestion in the left eye, corneal macula, the remaining condition was as preoperative. Treatment was then administered with 0.1% pranoprofen eye drops (4 times daily for 12 days) and 0.5% levofloxacin eye drops (4 times daily for 12 days). At the 20-day postoperative follow-up, signs showed mild eyelid edema and conjunctival congestion in the left eye, corneal macula, incomplete healing of the lateral limbus, the remaining findings were as preoperatively. The patient was treated with a compound formulation of tobramycin and dexamethasone eye drops (4 times daily for 5 days) and 40,000 IU recombinant human epidermal growth factor eye drops (4 times daily for 5 days). Approximately 1 month postoperatively, the patient underwent follow-up and complained of a foreign body sensation, photophobia, and tearing in the left eye. Examination showed mild swelling of the left eyelid, conjunctival congestion, and patchy macula in the temporal cornea, accompanied by partial epithelial defect. Fluorescent staining revealed a ring-shaped defect in the corneal epithelium. The remaining ocular conditions were the same as preoperatively, and the diagnosis was “left corneal epithelial injury.” The patient was treated with a compound formulation of tobramycin and dexamethasone eye drops (3 times daily for 4 days), 40,000 IU recombinant human epidermal growth factor eye drops (4 times daily for 4 days), and 5,000 IU vitamin A palmitate ophthalmic gel (3 times daily for 4 days). At the follow-up visit 3 days later, clinical signs showed no eyelid edema, conjunctival congestion, and corneal macula. Anterior segment photography showed a ring-shaped defect near the lateral canthal limbus of the cornea (Figures 1A–C), with the central cornea being transparent (Figures 1D–E). The remaining ocular conditions were the same as preoperatively, and the preliminary diagnosis was “left eye rodent corneal ulcer.”

**Figure 2:**
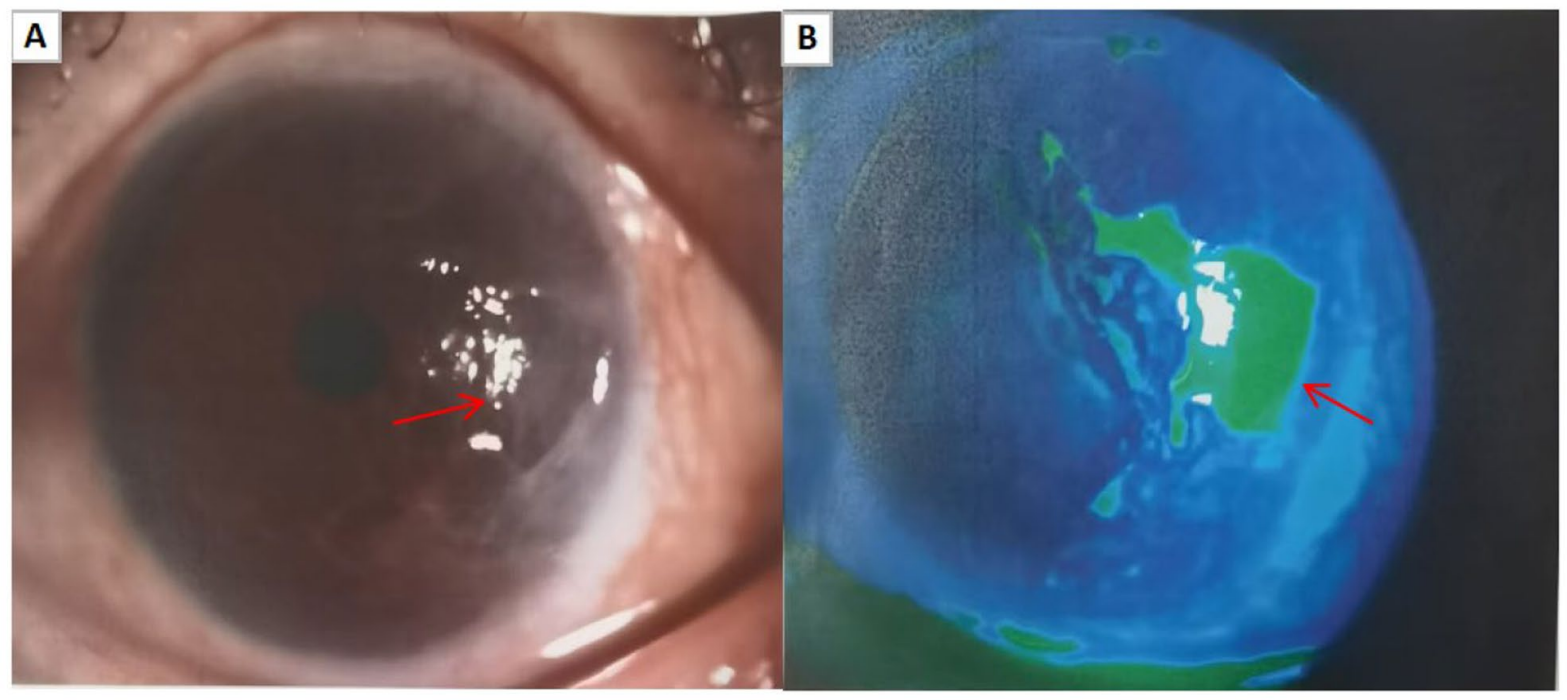
**(A,B)** Follow-up 3 days after immunotherapy in the left eye showed that anterior segment photography with fluorescein staining revealed a geographic superficial ulcer in the temporal cornea of the left eye.

**Figure 3:**
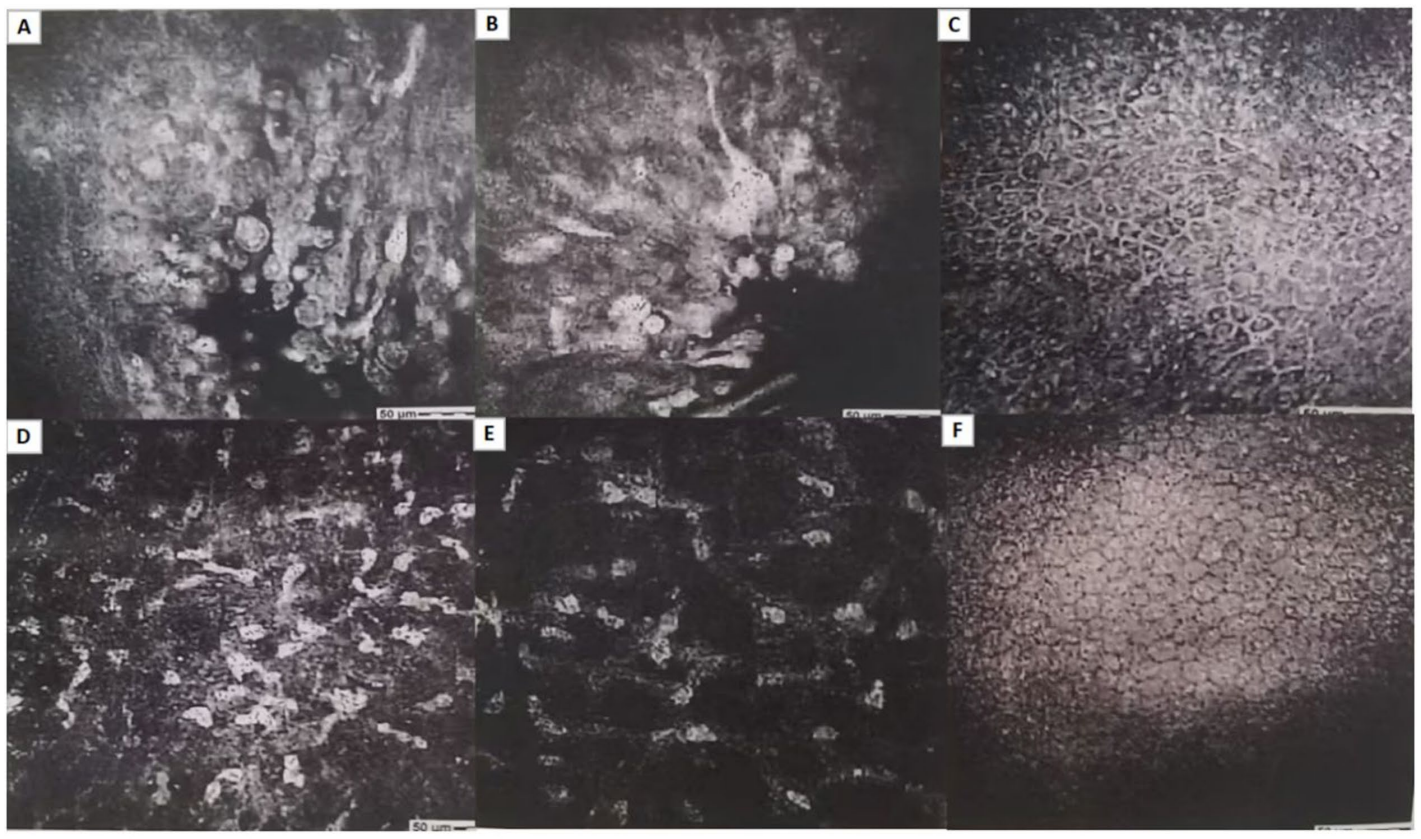
**(A–F)** Corneal confocal microscopy of the left eye revealed partial epithelial defect in the lesion area, accompanied by irregular punctate hyper-reflectivity and inflammatory cells, disappearance of sub-epithelial nerve fibers, activated Langerhans cells, and activated stromal cells with patchy irregular deposits in the surrounding area, and no significant changes in the corneal endothelial cells in the peripheral zone.

The patient presented again with the main complaint of “redness and pain in the left eye.” On examination, the external structures of the left eye were unremarkable, with conjunctival congestion and a large defect in the temporal cornea, the remaining examination findings were consistent with the pre-operative state. A possible autoimmune reaction was considered, and treatment was initiated with 0.1% tacrolimus eye drops (twice daily for 3 days), 21,000 IU recombinant bovine basic fibroblast growth factor gel (twice daily for 3 days), and 0.3% gatifloxacin eye gel (3 times daily for 3 days). The patient returned for a follow-up visit after 3 days of medication, and the subjective symptoms were reported as unchanged. Left eye vision was 0.15, intraocular pressure was 8.3 mmHg, and conjunctival congestion was observed. Slit-lamp photography of the anterior segment revealed a geographic superficial ulcer in the temporal cornea (Figures 2A,B), with no significant stromal infiltration observed. Corneal sensation testing (Cotton-tipped applicator method) indicated reduced corneal sensation in the left eye. Tear ELISA showed significantly elevated HSV-IgA levels. Corneal confocal microscopy of the left eye revealed partial epithelial defect in the lesion area, accompanied by irregular punctate hyper-reflectivity and inflammatory cells, disappearance of subepithelial nerve fibers, activated Langerhans cells and activated stromal cells with patchy irregular deposits in the surrounding area, and no significant changes in the corneal endothelial cells in the peripheral zone (Figures 3A–F). Based on the above clinical features, a preliminary diagnosis of “viral keratitis in the left eye” was made, followed by treatment with 0.15% ganciclovir ophthalmic gel (4 times daily for 7 days) and ganciclovir capsules (0.25 g, four capsules each time, three times daily, orally for 7 days).

**Figure 4:**
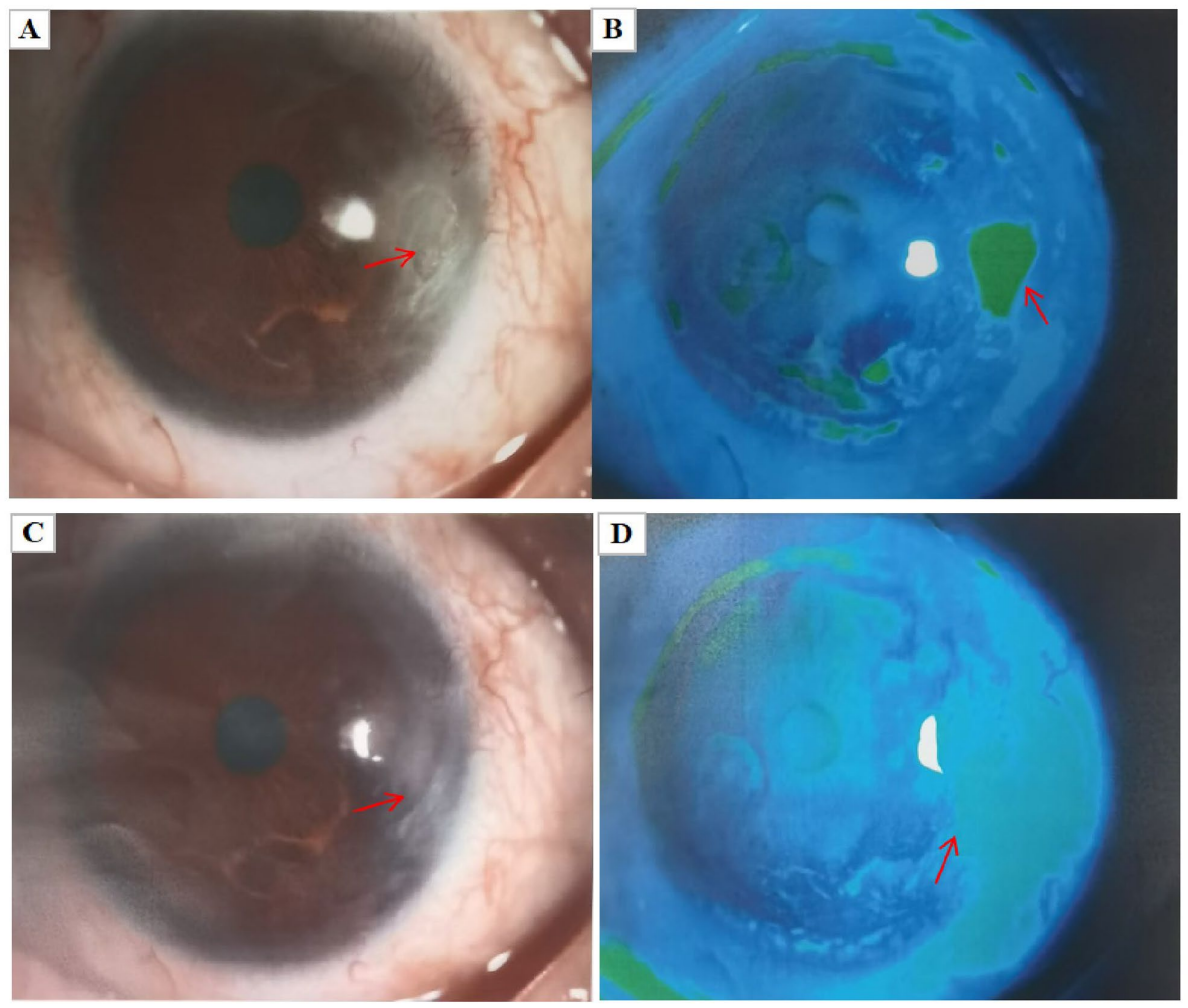
**(A,B)** Left eye keratitis follow-up after 1 week of medication, fluorescein staining slit-lamp photography showed that the corneal epithelium of the left eye had healed compared to before. **(C,D)** At the 2-week follow-up for left eye keratitis medication, a left eye temporal corneal macula was visible, and anterior segment photography with fluorescein staining showed that the corneal epithelium had completely healed.

During the follow-up visit 8 days after initiating antiviral treatment for left keratitis, the patient reported partial improvement in the symptoms of left eye redness and pain. On examination, left eye vision was 0.5 and intraocular pressure was 9.5 mmHg. Mild conjunctival congestion was observed on the temporal side. Anterior segment photography with fluorescein staining showed that the corneal epithelium had healed compared to the previous examination (Figures 4A,B). The regimen was continued with a reduced dose of ganciclovir capsules (0.25 g, 2 capsules each time, 3 times daily, orally for 2 weeks). Two weeks later, the patient returned for follow-up, reporting complete absence of symptoms. Examination showed a visual acuity of 0.6 and an IOP of 14.5 mmHg in the left eye. The external examination of the left eye was normal, with no significant conjunctival congestion. A temporal corneal macula was visible. Anterior segment photography with fluorescein staining indicated complete healing of the corneal epithelium (Figures 4C,D). Oral ganciclovir capsules were continued at a maintenance dose (0.25 g, 2 capsules each time, 3 times daily) and discontinued after 2 weeks. No recurrences were observed in the following 6 months. (Systemic antiviral treatment (7): ganciclovir: Oral 1 g/time, 3 times daily for 14 days; after clinical remission, oral 0.5 g/time, 3 times daily for a 2-month maintenance period. The specific timing for dosage reduction and discontinuation requires a comprehensive assessment based on the patient’s ocular and systemic conditions, confirming complete healing of corneal lesions and no signs of inflammatory recurrence).

This research protocol adheres to the principles of the Helsinki Declaration and has been approved by the Medical Ethics Committee of the Zhejiang Provincial Tongde Hospital, Affiliated to Zhejiang Chinese Medical University (College of Integrated Traditional Chinese and Western Medicine Clinical Medicine), Hangzhou, China (Approval No: Zhejiang Provincial Tongde Hospital Medical Ethics Committee 2,026,040-JY). All clinical data were sourced from the electronic medical record system and informed consent has been obtained from all patients.

## Discussion

This case is used to illustrate that corneal confocal microscopy has significant clinical importance in clarifying the etiology of corneal diseases.

Because in pterygium excision surgery it is necessary to excise hyperplastic conjunctival fibrous tissue, cauterize the sclera, and strip the pterygium tissue attached to the corneal surface, this leads to a large area of corneal wound. The main manifestation is the loss of corneal epithelium, Bowman’s membrane, and the epithelial basement membrane, as well as the discontinuity of stromal collagen. In the process of tissue repair, there is excessive proliferation of the corneal epithelium and vacuolation of basal epithelial cells. Coupled with the damage to nerve endings in the surgical area, metabolism and tear film formation are affected, which can easily lead to corneal ulcers (8). Age plays a certain role in the healing process, younger patients usually exhibit more vigorous tissue metabolism and a stronger inflammatory response, and are therefore more prone to excessive scar formation and neovascularisation, thus increasing the risk of recurrence. Conversely, the healing ability of older patients declines, which may lead to delayed graft integration (9, 10). Systemic diseases, such as diabetes, can damage microcirculation and immune function, increasing the risk of infection and delaying healing. Therefore, it can be inferred that elderly diabetic patients are more prone to develop corneal complications after pterygium excision surgery. Analysis of its causes may be as follows: Firstly, the blood-aqueous barrier function in patients with diabetes mellitus is already compromised, and a long-term hyperglycaemic state increases the glucose content in the aqueous humor, enhancing corneal endothelial permeability and reducing corneal endothelial cell function (11, 12); secondly, hyperglycaemia stimulates local capillaries in the ocular surface, activating the immune system and leading to elevated levels of inflammatory cytokines, which induces chronic inflammation, and multiple studies have confirmed that corneal ulcers following pterygium surgery are closely related to inflammation (13–15); thirdly, the presence of underlying diseases that affect the body’s internal homeostasis is also a significant factor increasing the risk of corneal ulceration; fourthly, patients with diabetes mellitus are more susceptible to intraoperative damage during pterygium surgery, which can easily lead to postoperative corneal edema or even ulcers.

According to the past medical history of the patient’s records, it can be determined that the patient currently has recurrent pterygium, and there have been studies reporting the association between the recurrence of pterygium and the coexistence of human papillomavirus (HPV) and herpes simplex virus. Detorakis et al. utilized polymerase chain reaction (PCR) technology to detect HSV in resected pterygium tissue, which is consistent with the previous research findings of Detorakis et al. (16) and Spandidos et al. (17). The results of the study indicate that herpes simplex virus-1 (HSV-1) was detected in all HSV-positive samples, and HPV-18 was detected in all HPV-positive samples. It has already been proposed that HPV and HSV fragments may coexist and may have a synergistic effect in the multi-step transformation process (18, 19). As the facts show, patients who are simultaneously detected with HSV and HPV are more common with postoperative recurrence and a history of conjunctivitis, indicating that the interaction between these viruses may affect the clinical characteristics of pterygium. In neither of the two studies was HSV detected in normal conjunctival specimens, which suggests that HSV may play a specific role in the pathogenesis of pterygium. In our study, the infiltrating lesion was located in the corneal area that had previously undergone pterygium excision. Multiple reports have provided strong evidence to support this view (20, 21). In previous epidemiological studies on HSV infection, most participants were elderly patients aged ≥55 years (22–24), consistent with the subjects in this study. This may be attributed to age-related changes in the immune system, which can lead to a decline in immune surveillance capabilities and increase susceptibility to herpes simplex virus reactivation. Elderly individuals often experience immunosenescence, a gradual decline in immune function that may impair the body’s ability to suppress latent herpes simplex virus infections. In addition, in this case, the patient with diabetes mellitus presented with persistent corneal epithelial defect. Research has demonstrated an association between diabetes and an increased incidence of HSK. This may be because hyperglycemia can damage the corneal epithelium, potentially increasing susceptibility to herpes simplex virus infection, thereby highlighting the urgent need for careful management of diabetes to mitigate its impact on HSK (22, 25).

HSK is a viral keratitis caused by HSV infection, and is one of the more common blinding corneal diseases in clinical practice. The etiology is divided into primary infection, latency and recurrent infection, and immune mechanisms. The inducing factors of HSK (26–29) include ocular factors (use of ocular medications, such as glucocorticoid eye drops; ocular surgery, laser treatment; trauma), systemic factors (systemic immunocompromised state caused by various reasons), and other factors (stress, fatigue, cold, ultraviolet radiation, and menstrual cycle, etc.). The main clinical symptoms include eye redness, eye pain, foreign body sensation, and varying degrees of photophobia, tearing, and decreased visual acuity. It is classified according to the site of corneal involvement as follows: 1. epithelial HSK; 2. stromal HSK; 3. endothelial HSK; 4. neurotrophic keratopathy. Among them, epithelial HSK is subdivided into corneal edema type, dendritic keratitis, geographic keratitis, and marginal keratitis based on different corneal signs. The clinical diagnosis of HSK is mainly based on medical history, inducing factors, and typical ocular signs. The differential diagnosis of HSK is crucial, as the clinical presentation of HSK is atypical and may overlap with other infectious and non-infectious keratitis, such as bacterial, fungal, and neurotrophic keratitis (30). In contrast, bacterial keratitis presents with similar early symptoms but is typically acute in onset and accompanied by purulent discharge. Patients with fungal keratitis typically exhibit corneal pain, redness, and ulcers, with the lesion edge exhibiting a spiky or feathery infiltration, and characteristic hyphal plaques and satellite lesions can be observed, with a prolonged course. Neurotrophic keratitis is a degenerative corneal disease caused by trigeminal nerve injury (such as from viral infection, tumors, or surgery), which can lead to dry eye, corneal epithelial defects or ulcers, corneal stromal melting and perforation, and diminished or absent corneal sensation (31). Mooren’s ulcer is characterized by severe pain that often does not correspond with clinical signs. The ulceration typically originates from the limbal region of the cornea within the palpebral fissure. The lesion borders are undermined, involving the superficial and intermediate stromal layers of the cornea. It is often covered by regenerated epithelium and infiltrated by neovascularization. The course of the disease is progressive (32). The treatment of HSK should be based on the specific clinical classification of HSK, followed by the use of corresponding standard methods. Among them, epithelial HSK is mainly treated with ocular antiviral treatment, and the use of glucocorticoids is generally contraindicated. Therefore, when host immunity decreases and under the influence of inducing factors, latent HSV-1 is activated, replicates within neuronal cells, and travels along the nerve fiber axoplasm to the innervated area and nearby tissues where it replicates in large quantities, releasing viral particles to cause a chronic inflammatory reaction (33).

*In vivo* confocal microscopy of the cornea: (1) To assist in evaluating the changes and injury repair of corneal tissues and cells after viral infection; (2) To understand the degree of inflammatory response and immunological changes in the diseased cornea; (3) To exclude other microbial infections such as fungi or Acanthamoeba (7). In patients with herpetic simplex virus keratitis, not only is the number of corneal nerve bundles significantly reduced, but they are also discontinuous and have multiple branchings. There is also a dendritic infiltration of a large number of Langerhans cells around the nerves, while the density of epithelial cells is decreased (34). In the lesion area of patients with fungal keratitis, fungal hyphae and spores can sometimes be observed, and an inflammatory cell infiltration predominantly composed of neutrophils can be seen. In the lesion area of patients with bacterial keratitis, bacterial bodies can be seen as highly reflective points with a diameter of 1-2 μm, and the infiltrated inflammatory cells are mainly polymorphonuclear white blood cells with a diameter of 8–10 μm. However, corneal confocal microscopy has certain limitations, such as being affected by the patient’s level of cooperation and the cornea’s transparency and dryness. If the patient has a high level of cooperation, and the cornea is without edema or dryness, it facilitates confocal microscopy examination (35).

In this case of an elderly patient with diabetes, surgical trauma as well as postoperative modulation of ocular immune response due to steroid use could both lead to the reactivation of HSK (36). In one study, an elderly female patient had no systemic diseases causing immunosuppression and did not use any systemic or topical steroids after pterygium excision, which differs from the case of the elderly male patient in this study. Therefore, Mahajan and Prasher (20) speculated that the HSK occurring after pterygium excision in that case was possibly caused solely by surgical trauma reactivating latent HSV infection. Previous research was based only on the use of antiviral treatment after fluorescein staining showed typical dendritic ulcers with terminal bulbs. In this study, the patient presented with persistent corneal epithelial defect, which were misdiagnosed as “rodent corneal ulcer” based on the morphological characteristics and location of the corneal ulcer. However, treatment with anti-immunosuppressive therapy was ineffective. Subsequently, given the evidence from corneal confocal microscopy, combined with the patient’s medical history and main clinical manifestations, empirical antiviral treatment was administered. The patient’s symptoms alleviated, and the epithelial defect healed, supporting the diagnosis of herpetic simplex virus keratitis in this case.

## Conclusion

For recurrent pterygium or post-excision patients developing early postoperative epithelial or stromal keratitis, consider HSV infection. Regardless of keratitis history, suspected cases warrant corneal fluorescein staining, in-vivo confocal microscopy, and other examinations to exclude HSV. Corneal confocal microscopy provides visual evidence of secondary viral infections following pterygium excision surgery, which is beneficial for early diagnosis and guiding treatment to achieve good visual prognosis.

## Data Availability

The original contributions presented in the study are included in the article/supplementary material, further inquiries can be directed to the corresponding author.
